# Identifying the educational needs of nurses caring for adults with congenital heart disease: A scoping review protocol

**DOI:** 10.1371/journal.pone.0343891

**Published:** 2026-07-10

**Authors:** Tommy Lin, Monica Parry, Tieghan Killackey

**Affiliations:** 1 Lawrence Bloomberg Faculty of Nursing, University of Toronto, Toronto, Ontario, Canada; 2 Peter Munk Cardiac Centre, Toronto General Hospital, University Health Network, Toronto, Ontario, Canada; Children’s Hospital of Los Angeles / Keck School of Medicine, UNITED STATES OF AMERICA

## Abstract

**Objective:**

The objective of this scoping review is to answer the question, “What is the existing evidence on nurses’ educational needs in caring for adults with congenital heart disease?” by identifying and summarizing research and grey literature on the educational needs of nurses who care for this population.

**Methods:**

The scoping review will use the Joanna Briggs Institute (JBI) methodology and Preferred Reporting Items for Systematic and Meta-Analyses extension for Scoping Reviews (PRISMA-ScR) for reporting. Five databases will be searched: Medline, Embase, CINAHL, ERIC, and Scopus. Grey Matters, Google, and thesis and dissertation databases will be used to identify relevant grey literature. All types of published articles and non-empirical research will be included with no temporal, geographical, or language restrictions. Two independent reviewers will screen titles and abstracts, engage in full-text screening, and perform data extraction in Covidence. Any disagreements will be resolved through a third reviewer.

**Results:**

The results of the scoping review will be analyzed using descriptive statistics and narrative analysis. The results will be reported using tables and figures. Findings will be disseminated through a manuscript and at conferences.

**Conclusion:**

The findings of this scoping review aim to inform future research and interventions on the nursing care of adults with congenital heart disease.

**Registration:**

The scoping review was registered on Open Science Framework (https://osf.io/4t5hr) on December 5, 2025.

## Introduction

Congenital heart diseases are structural and functional abnormalities of the heart, representing the most common congenital anomaly worldwide [1]. As a result of advances in detection, intervention, and long-term management, the survival to adulthood rate has increased to over 95% [2]. Consequently, there are now more adults living with congenital heart disease (ACHD) than children [2]. In Canada, the ACHD population was estimated at approximately 183,840 individuals in 2020, a 68% increase compared to 2007 [3].

ACHD present with unique care needs that differ from the typical adult cardiology population. The ACHD population represents a physiologically distinct cardiovascular group, characterized by structural defects, altered cardiac anatomy, and complex hemodynamic consequences resulting from prior surgical and catheter-based interventions [4–5]. Furthermore, ACHD experience an increased risk of acquired disorders such as heart failure, stroke, coronary artery disease, and atrial fibrillation [6–7]. Despite significant advances in the surgical and medical management of CHD, most interventions are palliative rather than curative [7]. As a result, ACHD often experience long-term cardiovascular complications and progressive disease over decades, necessitating lifelong surveillance and specialized ACHD expertise [7].

Advances in congenital cardiac surgery, interventional cardiology, and diagnostics have markedly improved survival, resulting in a growing and aging population of ACHD [3]. Beyond cardiovascular risk factors, ACHD face a variety of psychological and social challenges, many of which surround the transition into adult care. The transition from pediatric to adult cardiology care is a vulnerable period, with at least 30% of patients experiencing gaps in care or loss to follow-up [2]. During this transitional period, ACHD patients are managing developmental and psychosocial maturation, which can complicate self-management and navigation in the adult healthcare system [2]. Concurrently, ACHD are also undergoing identity formation and shifting social roles, which can contribute to psychological distress affecting transitions in education, employment, and relationships [2]. This is supported by Pelosi et al. [8] longitudinal cohort study, which showed lower educational level, occupational level, and employment rates in ACHD compared to the general population. Finally, the ACHD population also experiences an increased risk of anxiety, depression, and attention problems [2]. A recent meta-analysis [9] further supports the ongoing emotional stressors associated with living with CHD, highlighting the psychological burden of lifelong disease management in this population.

Despite the recognition of the need for specialized ACHD care, nursing education in this area has not evolved at the same pace as medical education [4,10]. Physicians have access to structured postgraduate pathways, such as cardiology residencies and dedicated adult congenital heart disease fellowships, yet no equivalent specialized education exists for nurses [3]. Moreover, pediatric nurses, particularly those working in pediatric cardiac and cardiac intensive care settings, develop specialized expertise in congenital heart disease through unit-based education, clinical exposure, and simulation-based training [11–13]. In contrast, adult cardiovascular nursing education is largely generalist and focused on nursing management of acquired heart disease. This gap in education presents as a critical issue, as nurses provide ongoing care and support to ACHD patients in both inpatient and outpatient settings. This can lead to inconsistencies in the quality of care and missed opportunities for patient education, self-management, and psychosocial support.

Finally, there is existing literature that examines the educational needs and gaps among nurses caring for this population. For example, earlier literature identified systematic gaps in ACHD education, including limited formal education, inadequate resources, and a mismatch between patient needs and workforce preparedness [14]. A cross-sectional study by Griffiths et al. [15] further supports this, as nurses reported low confidence, limited knowledge, and insufficient training in caring for ACHD. However, a preliminary search of the literature did not identify any existing scoping or systematic reviews on this topic. Therefore, identifying existing educational needs for nurses in caring for ACHD is essential to guide future nursing curriculum development and improve care delivery for this growing population.

This scoping review aims to answer the question, “What is the existing evidence on nurses’ educational needs in caring for ACHD?” by identifying and summarizing research and grey literature on the educational needs of nurses who care for this population.

## Methods

The scoping review will follow the Joanna Briggs Institute (JBI) methodology [16] and use the Preferred Reporting Items for Systematic and Meta-Analyses extension for Scoping Reviews (PRISMA-ScR) for reporting the scoping review [17]. The scoping review protocol was developed using the Preferred Reporting Items for Systematic Review and Meta-Analysis Protocols (PRISMA-P); please see S1 File: PRISMA-P Checklist [18]. This protocol was further informed by Arksey and O’Malley’s [19] five-stage framework for scoping reviews, which includes: (1) identifying the research question, (2) identifying relevant studies, (3) study selection, (4) data charting, and (5) collating, summarizing and reporting the results. The protocol for this scoping review has been registered with the Open Science Framework (https://osf.io/4t5hr) on December 5, 2025.

### Step 1: Identify the research question

The research question was created in accordance with the JBI using the Population, Concept, and Context (PCC) framework [17]. The primary research question is as follows:

What is the existing evidence on nurses’ educational needs in caring for ACHD?

### Step 2: Identify relevant studies

#### Eligibility criteria.

The inclusion criteria and scope of this review were structured based on the PCC framework [17].

#### Population.

The review focuses on nurses involved in the care of ACHD. For the purposes of this review, nurses will include regulated nursing designations such as Registered Nurses (RN), Registered Practical Nurses (RPN)/Licensed Practical Nurses (LPN), and Nurse Practitioners (NP), whose practice roles may include advanced practice nurses, clinical nurse specialists, cardiac nurses, congenital/ACHD nurses, and nurse educators with a focus on nurses’ learning needs.

#### Concept.

The core concept of this review is the educational needs for nursing care of ACHD. Educational needs may include knowledge gaps, skill deficits, competency frameworks, learning needs assessments, training program needs, perceived preparedness, barriers/facilitators to education.

#### Context.

The context includes caring for ACHD across various healthcare settings. This can include ACHD outpatient clinics, adult cardiology wards, congenital specialty centers, community/primary care, emergency care, intensive care, and transition services when focused on adult care.

#### Information sources and search strategy.

A health sciences librarian was consulted to create the search strategy, which consisted of a combination of keywords and index terms that focused on the population of ACHD and the concept of interest, which is nursing education. A complete literature search will be conducted using five databases: Medline, Embase, CINAHL, ERIC, and Scopus. The initial search will be conducted in Medline (Table 1). The Medline search strategy will then be translated into the other databases (Embase, CINAHL, ERIC, and Scopus) using the appropriate controlled vocabulary and verified with the health sciences librarian (S2 File). Furthermore, a customized Google Scholar search will be used to search for grey literature, restricting the results to the first 10 pages, and/or until results are no longer relevant [16]. In addition, grey literature sources, such as Grey Matters, and thesis and dissertation databases, will be reviewed to identify relevant literature [16].

**Table 1:** Medline search strategy.

#	Query	Results from 15 Apr 2026
1	exp Heart Defects, Congenital/	179,957
2	Heart Diseases/cn [Congenital]	749
3	(((congenital or neonat* or pediatric or structural or cyanotic or acyanotic) adj3 (heart or cardiac or coronary or septal* or aortopulmonary or aorticopulmonary or atrial or ventricular or intraventricular) adj3 (defect* or disease* or abnormal* or anomal* or malform* or condition* or care)) or (single ventricle defect* or biventricular defect*)).tw,kf.	73,989
4	((atrial or ventricular or atrioventricular) adj3 septal adj3 defect).tw,kf.	24,852
5	(pulmonary adj2 (atresia or regurgitation or stenosis or prolapse)).tw,kf.	14,073
6	(tricuspid adj2 (atresia or regurgitation or stenosis or prolapse)).tw,kf.	12,809
7	Eisenmenger syndrome.tw,kf.	962
8	(neonatal adj3 (cardiopath* or cardiomyopath*)).tw,kf.	182
9	(aort* adj2 (coarctation* or narrow* or stenosis)).tw,kf.	38,526
10	(bicuspid adj3 aort* adj3 valve*).tw,kf.	5,730
11	(persistent adj3 truncus adj3 arteriosus).tw,kf.	420
12	(truncus adj3 arteriosus adj3 communi*).tw,kf.	178
13	(endocardi* adj3 cushion* adj3 defect*).tw,kf.	524
14	(atrioventricular adj3 (canal* or cushion*)).tw,kf.	1,886
15	(aortopulmonary adj2 window*).tw,kf.	757
16	(common adj3 arter* adj3 trunk*).tw,kf.	667
17	(persistent adj3 ostium adj3 primum*).tw,kf.	14
18	(patent adj3 oval? adj3 foramen*).tw,kf.	7,304
19	(lutembacher* adj2 syndrome*).tw,kf.	211
20	(double adj3 outlet* adj3 right adj3 ventricle*).tw,kf.	2,168
21	(taussig adj3 bing adj3 anomal*).tw,kf.	181
22	(hypoplastic adj3 left adj3 heart* adj3 syndrome*).tw,kf.	4,091
23	(fallot* adj2 (tetralog* or tetrad* or syndrome*)).tw,kf.	12,433
24	(transposition adj3 great adj3 (arter* or vessel*)).tw,kf.	7,911
25	(levotransposition* adj3 great adj3 (arter* or vessel*)).tw,kf.	9
26	(levo adj2 tga*).tw,kf.	12
27	L-TGA.tw,kf.	56
28	(dextrotransposition* adj3 great adj3 (arter* or vessel*)).tw,kf.	53
29	(dextro adj2 tga*).tw,kf.	25
30	D-TGA.tw,kf.	485
31	(congenital* adj3 corrected adj3 (transposition* or tga*)).tw,kf.	1,175
32	CC-TGA.tw,kf.	61
33	(left adj3 right adj3 shunt*).tw,kf.	7,826
34	(paten* adj3 atrioventricular adj3 canal*).tw,kf.	24
35	(cyanotic adj3 (cardia* or heart?) adj3 (disease* or defect*)).tw,kf.	2,868
36	(myocardial adj2 bridg*).tw,kf.	1,651
37	(single adj2 heart? adj2 ventricle?).tw,kf.	437
38	((monoventricular or univentricular) adj2 heart?).tw,kf.	1,417
39	(cor adj2 monoventricolare).tw,kf.	0
40	(cor adj2 triloculare adj4 (biatrium or biabriatum or biatriorum)).tw,kf.	5
41	(ventricular adj2 (non-compaction or noncompaction)).tw,kf.	2,219
42	(cardiomyopath* adj3 (non-compaction or noncompaction)).tw,kf.	987
43	(congenital adj2 (heart? or cardia*) adj2 block*).tw,kf.	1,316
44	exp Education, Nursing/	94,419
45	exp Education, Nursing, Continuing/	24,024
46	Nurse Clinicians/ed [Education]	1,894
47	(((nurs* or clinical) adj4 (educat* or train* or learn* or knowledg*)) or professional development or continuing educat* or ((learning or education* or knowledge) adj4 (need* or gap* or requirement* or deficien* or improv*)) or competenc* or (skill* adj3 train*) or (understanding adj3 improv*)).tw,kf.	589,567
48	or/1–43 [**congenital heart disease]	273,760
49	or/44–47 [**nursing education]	641,759
50	48 and 49	2,490

#### Types of sources.

The scoping review will include all types of published studies with quantitative, qualitative, or mixed-method designs. In addition, non-empirical research, such as grey literature, literature reviews, editorials, and opinion papers, will be included. No geographical, language, or temporal limitations will be set for this scoping review in order to ensure a comprehensive capture of all relevant literature on this topic. If relevant non-English articles are identified, a professional translator proficient in the respective language will be engaged to ensure accurate translation.

### Step 3: Study selection

The database results will be exported to Covidence to remove any duplicate titles and abstracts [20]. In accordance with JBI methodology, the remaining titles and abstracts will undergo pilot screening in which two reviewers will review a random sample of 25 studies [16]. The authors will meet to compare the included and excluded studies, ensuring a 75% consensus minimum is reached before full screening proceeds [16]. Any changes to the inclusion criteria will be made iteratively and disclosed in the methodology of the full scoping review [16]. After pilot testing, two independent reviewers will screen titles and abstracts using the Covidence software. Any disagreements will be resolved by a third reviewer in Covidence. Next, full texts will be independently reviewed by two reviewers according to the inclusion criteria. A third reviewer will resolve any conflict during full-text selection. All articles will be retrieved from either the University of Toronto library or through the Interlibrary Loan system (https://onesearch.library.utoronto.ca/request-items-other-institutions-ill). A PRISMA 2020 flow diagram will be generated using Covidence, detailing the number of studies included and excluded in the final scoping review. Finally, the reference lists of all included studies will be screened to identify any additional relevant sources that may have been missed during the database search.

### Step 4: Charting the data

The data will be extracted in Covidence by two independent reviewers and any disagreements will be resolved through a third reviewer. Before full extraction, two reviewers will pilot the extraction form on two sources to ensure all relevant results are extracted and consistency is established [16]. The extraction form will chart the following key information: authors, year, country, study aim, nurse population, setting, methodology, educational needs identified, and findings relevant to the purpose of the scoping review. The final results will be exported into an Excel spreadsheet for authors to conduct data analysis.

In addition, a quality assessment will be performed of the included studies using the Mixed Methods Appraisal Tool (MMAT) version 2018 [21–22]. The MMAT is a tool that will be used to assess quantitative, qualitative and mixed-methods studies [21–22], which aligns well with the review’s inclusion criteria. The quality assessment will be performed concurrently with the data extraction by two reviewers independently. Any disagreements will be solved by a third reviewer. However, no study will be excluded from the review based on the quality assessment.

### Step 5: Collating, summarizing, and reporting the results

The reporting of results will follow the PRISMA-ScR to ensure transparency and systematic review of the scoping review findings [17]. Numerical and descriptive statistics will be used to describe the overall number of studies included, sample size, geographic location, and methodology. Furthermore, qualitative data findings will be synthesized and presented through a narrative description, highlighting key themes related to nurses’ educational needs in caring for ACHD. Tables and figures will be used to represent the results of the scoping review and the quality assessment of studies.

## Conclusion

This scoping review protocol provides a methodology overview for a scoping review aimed at identifying and summarizing the existing evidence on nurses’ educational needs in caring for ACHD. This information will help inform future research and educational interventions on the nursing care of ACHD.

## Supporting information

S1 Table Medline search strategy. (DOCX)

S1 File PRISMA-P Checklist. (DOCX)

S2 File Search strategy for Embase, CINAHL, ERIC, and Scopus. (DOCX)

S3 File Prisma Flow Diagram. (DOCX)
