# Supplementary material for: Identifying the educational needs of nurses caring for adults with congenital heart disease: A scoping review protocol
Source: PLoS One. 2026 Jul 10;21(7):e0343891. doi: 10.1371/journal.pone.0343891 (PMC13353937; doi:10.1371/journal.pone.0343891)
Supplement: S1 Table — (DOCX) [file pone.0343891.s001.docx]

**Table 1. Medline search strategy.**

| **#** | **Query** | **Results from 15 Apr 2026** |
| --- | --- | --- |
| 1 | exp Heart Defects, Congenital/ | 179,957 |
| 2 | Heart Diseases/cn [Congenital] | 749 |
| 3 | (((congenital or neonat* or pediatric or structural or cyanotic or acyanotic) adj3 (heart or cardiac or coronary or septal* or aortopulmonary or aorticopulmonary or atrial or ventricular or intraventricular) adj3 (defect* or disease* or abnormal* or anomal* or malform* or condition* or care)) or (single ventricle defect* or biventricular defect*)).tw,kf. | 73,989 |
| 4 | ((atrial or ventricular or atrioventricular) adj3 septal adj3 defect).tw,kf. | 24,852 |
| 5 | (pulmonary adj2 (atresia or regurgitation or stenosis or prolapse)).tw,kf. | 14,073 |
| 6 | (tricuspid adj2 (atresia or regurgitation or stenosis or prolapse)).tw,kf. | 12,809 |
| 7 | Eisenmenger syndrome.tw,kf. | 962 |
| 8 | (neonatal adj3 (cardiopath* or cardiomyopath*)).tw,kf. | 182 |
| 9 | (aort* adj2 (coarctation* or narrow* or stenosis)).tw,kf. | 38,526 |
| 10 | (bicuspid adj3 aort* adj3 valve*).tw,kf. | 5,730 |
| 11 | (persistent adj3 truncus adj3 arteriosus).tw,kf. | 420 |
| 12 | (truncus adj3 arteriosus adj3 communi*).tw,kf. | 178 |
| 13 | (endocardi* adj3 cushion* adj3 defect*).tw,kf. | 524 |
| 14 | (atrioventricular adj3 (canal* or cushion*)).tw,kf. | 1,886 |
| 15 | (aortopulmonary adj2 window*).tw,kf. | 757 |
| 16 | (common adj3 arter* adj3 trunk*).tw,kf. | 667 |
| 17 | (persistent adj3 ostium adj3 primum*).tw,kf. | 14 |
| 18 | (patent adj3 oval? adj3 foramen*).tw,kf. | 7,304 |
| 19 | (lutembacher* adj2 syndrome*).tw,kf. | 211 |
| 20 | (double adj3 outlet* adj3 right adj3 ventricle*).tw,kf. | 2,168 |
| 21 | (taussig adj3 bing adj3 anomal*).tw,kf. | 181 |
| 22 | (hypoplastic adj3 left adj3 heart* adj3 syndrome*).tw,kf. | 4,091 |
| 23 | (fallot* adj2 (tetralog* or tetrad* or syndrome*)).tw,kf. | 12,433 |
| 24 | (transposition adj3 great adj3 (arter* or vessel*)).tw,kf. | 7,911 |
| 25 | (levotransposition* adj3 great adj3 (arter* or vessel*)).tw,kf. | 9 |
| 26 | (levo adj2 tga*).tw,kf. | 12 |
| 27 | L-TGA.tw,kf. | 56 |
| 28 | (dextrotransposition* adj3 great adj3 (arter* or vessel*)).tw,kf. | 53 |
| 29 | (dextro adj2 tga*).tw,kf. | 25 |
| 30 | D-TGA.tw,kf. | 485 |
| 31 | (congenital* adj3 corrected adj3 (transposition* or tga*)).tw,kf. | 1,175 |
| 32 | CC-TGA.tw,kf. | 61 |
| 33 | (left adj3 right adj3 shunt*).tw,kf. | 7,826 |
| 34 | (paten* adj3 atrioventricular adj3 canal*).tw,kf. | 24 |
| 35 | (cyanotic adj3 (cardia* or heart?) adj3 (disease* or defect*)).tw,kf. | 2,868 |
| 36 | (myocardial adj2 bridg*).tw,kf. | 1,651 |
| 37 | (single adj2 heart? adj2 ventricle?).tw,kf. | 437 |
| 38 | ((monoventricular or univentricular) adj2 heart?).tw,kf. | 1,417 |
| 39 | (cor adj2 monoventricolare).tw,kf. | 0 |
| 40 | (cor adj2 triloculare adj4 (biatrium or biabriatum or biatriorum)).tw,kf. | 5 |
| 41 | (ventricular adj2 (non-compaction or noncompaction)).tw,kf. | 2,219 |
| 42 | (cardiomyopath* adj3 (non-compaction or noncompaction)).tw,kf. | 987 |
| 43 | (congenital adj2 (heart? or cardia*) adj2 block*).tw,kf. | 1,316 |
| 44 | exp Education, Nursing/ | 94,419 |
| 45 | exp Education, Nursing, Continuing/ | 24,024 |
| 46 | Nurse Clinicians/ed [Education] | 1,894 |
| 47 | (((nurs* or clinical) adj4 (educat* or train* or learn* or knowledg*)) or professional development or continuing educat* or ((learning or education* or knowledge) adj4 (need* or gap* or requirement* or deficien* or improv*)) or competenc* or (skill* adj3 train*) or (understanding adj3 improv*)).tw,kf. | 589,567 |
| 48 | or/1-43 [**congenital heart disease] | 273,760 |
| 49 | or/44-47 [**nursing education] | 641,759 |
| 50 | 48 and 49 | 2,490 |
