# Supplementary material for: Identifying the educational needs of nurses caring for adults with congenital heart disease: A scoping review protocol
Source: PLoS One. 2026 Jul 10;21(7):e0343891. doi: 10.1371/journal.pone.0343891 (PMC13353937; doi:10.1371/journal.pone.0343891)
Supplement: S2 File — (DOCX) [file pone.0343891.s003.docx]

**Embase Search Strategy**

| **#** | **Query** | **Results from 15 Apr 2026** |
| --- | --- | --- |
| 1 | exp Heart Defects, Congenital/ | 215,246 |
| 2 | Heart Diseases/cn [Congenital] | 933 |
| 3 | (((congenital or neonat* or pediatric or structural or cyanotic or acyanotic) adj3 (heart or cardiac or coronary or septal* or aortopulmonary or aorticopulmonary or atrial or ventricular or intraventricular) adj3 (defect* or disease* or abnormal* or anomal* or malform* or condition* or care)) or (single ventricle defect* or biventricular defect*)).tw,kf. | 119,682 |
| 4 | ((atrial or ventricular or atrioventricular) adj3 septal adj3 defect).tw,kf. | 38,541 |
| 5 | (pulmonary adj2 (atresia or regurgitation or stenosis or prolapse)).tw,kf. | 23,201 |
| 6 | (tricuspid adj2 (atresia or regurgitation or stenosis or prolapse)).tw,kf. | 23,336 |
| 7 | Eisenmenger [syndrome.tw](http://syndrome.tw/),kf. | 1,731 |
| 8 | (neonatal adj3 (cardiopath* or cardiomyopath*)).tw,kf. | 272 |
| 9 | (aort* adj2 (coarctation* or narrow* or stenosis)).tw,kf. | 64,350 |
| 10 | (bicuspid adj3 aort* adj3 valve*).tw,kf. | 9,569 |
| 11 | (persistent adj3 truncus adj3 arteriosus).tw,kf. | 592 |
| 12 | (truncus adj3 arteriosus adj3 communi*).tw,kf. | 264 |
| 13 | (endocardi* adj3 cushion* adj3 defect*).tw,kf. | 787 |
| 14 | (atrioventricular adj3 (canal* or cushion*)).tw,kf. | 2,645 |
| 15 | (aortopulmonary adj2 window*).tw,kf. | 1,008 |
| 16 | (common adj3 arter* adj3 trunk*).tw,kf. | 1,074 |
| 17 | (persistent adj3 ostium adj3 primum*).tw,kf. | 44 |
| 18 | (patent adj3 oval? adj3 foramen*).tw,kf. | 12,309 |
| 19 | (lutembacher* adj2 syndrome*).tw,kf. | 336 |
| 20 | (double adj3 outlet* adj3 right adj3 ventricle*).tw,kf. | 3,282 |
| 21 | (taussig adj3 bing adj3 anomal*).tw,kf. | 275 |
| 22 | (hypoplastic adj3 left adj3 heart* adj3 syndrome*).tw,kf. | 6,427 |
| 23 | (fallot* adj2 (tetralog* or tetrad* or syndrome*)).tw,kf. | 19,754 |
| 24 | (transposition adj3 great adj3 (arter* or vessel*)).tw,kf. | 12,315 |
| 25 | (levotransposition* adj3 great adj3 (arter* or vessel*)).tw,kf. | 12 |
| 26 | (levo adj2 tga*).tw,kf. | 17 |
| 27 | [L-TGA.tw](http://l-tga.tw/),kf. | 147 |
| 28 | (dextrotransposition* adj3 great adj3 (arter* or vessel*)).tw,kf. | 75 |
| 29 | (dextro adj2 tga*).tw,kf. | 26 |
| 30 | [D-TGA.tw](http://d-tga.tw/),kf. | 989 |
| 31 | (congenital* adj3 corrected adj3 (transposition* or tga*)).tw,kf. | 1,802 |
| 32 | [CC-TGA.tw](http://cc-tga.tw/),kf. | 134 |
| 33 | (left adj3 right adj3 shunt*).tw,kf. | 14,315 |
| 34 | (paten* adj3 atrioventricular adj3 canal*).tw,kf. | 26 |
| 35 | (cyanotic adj3 (cardia* or heart?) adj3 (disease* or defect*)).tw,kf. | 4,827 |
| 36 | (myocardial adj2 bridg*).tw,kf. | 2,608 |
| 37 | (single adj2 heart? adj2 ventricle?).tw,kf. | 789 |
| 38 | ((monoventricular or univentricular) adj2 heart?).tw,kf. | 2,246 |
| 39 | (cor adj2 monoventricolare).tw,kf. | 0 |
| 40 | (cor adj2 triloculare adj4 (biatrium or biabriatum or biatriorum)).tw,kf. | 10 |
| 41 | (ventricular adj2 (non-compaction or noncompaction)).tw,kf. | 3,641 |
| 42 | (cardiomyopath* adj3 (non-compaction or noncompaction)).tw,kf. | 1,798 |
| 43 | (congenital adj2 (heart? or cardia*) adj2 block*).tw,kf. | 2,095 |
| 44 | exp Education, Nursing/ | 105,790 |
| 45 | exp Education, Nursing, Continuing/ | 105,790 |
| 46 | exp clinical nurse specialist/ | 3,793 |
| 47 | (((nurs* or clinical) adj4 (educat* or train* or learn* or knowledg*)) or professional development or continuing educat* or ((learning or education* or knowledge) adj4 (need* or gap* or requirement* or deficien* or improv*)) or competenc* or (skill* adj3 train*) or (understanding adj3 improv*)).tw,kf. | 778,976 |
| 48 | or/1-43 [**congenital heart disease] | 374,582 |
| 49 | or/44-47 [**nursing education] | 840,845 |
| 50 | 48 and 49 | 4,174 |

**CINAHL Search Strategy**

| **#** | **Query** | **Results from 15 Apr 2026** |
| --- | --- | --- |
| S1 | (MH "Heart Defects, Congenital") | 13,662 |
| S2 | XB (((congenital or neonat* or pediatric or structural or cyanotic or acyanotic) N3 (heart or cardiac or coronary or septal* or aortopulmonary or aorticopulmonary or atrial or ventricular or intraventricular) N3 (defect* or disease* or abnormal* or anomal* or malform* or condition* or care)) or (single ventricle defect* or biventricular defect*)) | 15,690 |
| S3 | XB ((atrial or ventricular or atrioventricular) N3 septal N3 defect) | 4,431 |
| S4 | XB (pulmonary N2 (atresia or regurgitation or stenosis or prolapse)) | 2,088 |
| S5 | XB (tricuspid N2 (atresia or regurgitation or stenosis or prolapse)) | 2,615 |
| S6 | XB Eisenmenger syndrome | 308 |
| S7 | XB (neonatal N3 (cardiopath* or cardiomyopath*)) | 32 |
| S8 | XB (aort* N2 (coarctation* or narrow* or stenosis)) | 7,945 |
| S9 | XB (bicuspid N3 aort* N3 valve*) | 1,245 |
| S10 | XB (persistent N3 truncus N3 arteriosus) | 30 |
| S11 | XB (truncus N3 arteriosus N3 communi*) | 13 |
| S12 | XB (endocardi* N3 cushion* N3 defect*) | 37 |
| S13 | XB (atrioventricular N3 (canal* or cushion*)) | 119 |
| S14 | XB (aortopulmonary N2 window*) | 138 |
| S15 | XB (common N3 arter* N3 trunk*) | 129 |
| S16 | XB (ostium N3 primum N3 defect*) | 17 |
| S17 | XB (patent N3 oval? N3 foramen*) | 9 |
| S18 | XB (lutembacher* N2 syndrome*) | 22 |
| S19 | XB (double N3 outlet* N3 right N3 ventricle*) | 218 |
| S20 | XB (taussig N3 bing N3 anomal*) | 15 |
| S21 | XB (hypoplastic N3 left N3 heart* N3 syndrome*) | 841 |
| S22 | XB (fallot* N2 (tetralog* or tetrad* or syndrome*)) | 2,128 |
| S23 | XB (transposition N3 great N3 (arter* or vessel*)) | 1,277 |
| S24 | XB (levotransposition* N3 great N3 (arter* or vessel*)) | 0 |
| S25 | XB (levo N2 tga*) | 4 |
| S26 | XB L-TGA | 18 |
| S27 | XB (dextrotransposition* N3 great N3 (arter* or vessel*)) | 13 |
| S28 | XB (dextro N2 tga*) | 3 |
| S29 | XB D-TGA | 105 |
| S30 | XB (congenital* N3 corrected N3 (transposition* or tga*)) | 274 |
| S31 | XB CC-TGA | 13 |
| S32 | XB (left N3 right N3 shunt*) | 1,272 |
| S33 | XB (paten* N3 atrioventricular N3 canal*) | 1 |
| S34 | XB (cyanotic N3 (cardia* or heart?) N3 (disease* or defect*)) | 498 |
| S35 | XB (myocardial N2 bridg*) | 384 |
| S36 | XB (single N2 heart? N2 ventricle?) | 201 |
| S37 | XB ((monoventricular or univentricular) N2 heart?) | 173 |
| S38 | XB (cor N2 monoventricolare) | 0 |
| S39 | XB (cor N2 triloculare N4 (biatrium or biabriatum or biatriorum)) | 0 |
| S40 | XB (ventricular N2 (non-compaction or noncompaction)) | 657 |
| S41 | XB (cardiomyopath* N3 (non-compaction or noncompaction)) | 337 |
| S42 | XB (congenital N2 (heart? or cardia*) N2 block*) | 276 |
| S43 | (MH "Education, Nursing") | 57,348 |
| S44 | (MH "Education, Nursing, Continuing") | 15,944 |
| S45 | (MH "Clinical Nurse Specialists/ED") | 464 |
| S46 | XB ((((nurs* or clinical) N4 (educat* or train* or learn* or knowledg*)) or professional development or continuing educat* or ((learning or education* or knowledge) N4 (need* or gap* or requirement* or deficien* or improv*)) or competenc* or (skill* N3 train*) or (understanding N3 improv*))) | 301,539 |
| S47 | S1 OR S2 OR S3 OR S4 OR S5 OR S6 OR S7 OR S8 OR S9 OR S10 OR S11 OR S12 OR S13 OR S14 OR S15 OR S16 OR S17 OR S18 OR S19 OR S20 OR S21 OR S22 OR S23 OR S24 OR S25 OR S26 OR S27 OR S28 OR S29 OR S30 OR S31 OR S32 OR S33 OR S34 OR S35 OR S36 OR S37 OR S38 OR S39 OR S40 OR S41 OR S42 | 41,272 |
| S48 | S43 OR S44 OR S45 OR S46 | 342,269 |
| S49 | S47 AND S48 | 591 |

**ERIC Search Strategy**

| **#** | **Query** | **Results from 15 Apr 2026** |
| --- | --- | --- |
| S1 | title(((congenital or neonat* or pediatric or structural or cyanotic or acyanotic) N/3 (heart or cardiac or coronary or septal* or aortopulmonary or aorticopulmonary or atrial or ventricular or intraventricular) N/3 (defect* or disease* or abnormal* or anomal* or malform* or condition* or care)) or (single ventricle defect* or biventricular defect*)) | 29 |
| S2 | title((atrial or ventricular or atrioventricular) N/3 septal N/3 defect) | 0 |
| S3 | title(pulmonary N/2 (atresia or regurgitation or stenosis or prolapse)) | 0 |
| S4 | title(tricuspid N/2 (atresia or regurgitation or stenosis or prolapse)) | 0 |
| S5 | title(Eisenmenger syndrome) | 0 |
| S6 | title(neonatal N/3 (cardiopath* or cardiomyopath*)) | 0 |
| S7 | title(aort* N/2 (coarctation* or narrow* or stenosis)) | 1 |
| S8 | title(bicuspid N/3 aort* N/3 valve*) | 0 |
| S9 | title(persistent N/3 truncus N/3 arteriosus) | 0 |
| S10 | title(truncus N/3 arteriosus N/3 communi*) | 0 |
| S11 | title(endocardi* N/3 cushion* N/3 defect*) | 0 |
| S12 | title(atrioventricular N/3 (canal* or cushion*)) | 0 |
| S13 | title(aortopulmonary N/2 window*) | 0 |
| S14 | title(common N/3 arter* N/3 trunk*) | 0 |
| S15 | title(persistent N/3 ostium N/3 primum*) | 0 |
| S16 | title(patent N/3 oval? N/3 foramen*) | 0 |
| S17 | title(lutembacher* N/2 syndrome*) | 0 |
| S18 | title(double N/3 outlet* N/3 right N/3 ventricle*) | 0 |
| S19 | title(taussig N/3 bing N/3 anomal*) | 0 |
| S20 | title(hypoplastic N/3 left N/3 heart* N/3 syndrome*) | 0 |
| S21 | title(fallot* N/2 (tetralog* or tetrad* or syndrome*)) | 1 |
| S22 | title(transposition N/3 great N/3 (arter* or vessel*)) | 1 |
| S23 | title(levotransposition* N/3 great N/3 (arter* or vessel*)) | 0 |
| S24 | title(levo N/2 tga*) | 0 |
| S25 | title(L-TGA) | 0 |
| S26 | title(dextrotransposition* N/3 great N/3 (arter* or vessel*)) | 0 |
| S27 | title(dextro N/2 tga*) | 0 |
| S28 | title(D-TGA) | 0 |
| S29 | title(congenital* N/3 corrected N/3 (transposition* or tga*)) | 0 |
| S30 | title(CC-TGA) | 0 |
| S31 | title(left N/3 right N/3 shunt*) | 0 |
| S32 | title(paten* N/3 atrioventricular N/3 canal*) | 0 |
| S33 | title(cyanotic N/3 (cardia* or heart?) N/3 (disease* or defect*)) | 0 |
| S34 | title(myocardial N/2 bridg*) | 0 |
| S35 | title(single N/2 heart? N/2 ventricle?) | 0 |
| S36 | title((monoventricular or univentricular) N/2 heart?) | 0 |
| S37 | title(cor N/2 monoventricolare) | 0 |
| S38 | title(cor N/2 triloculare N/4 (biatrium or biabriatum or biatriorum)) | 0 |
| S39 | title(ventricular N/2 (non-compaction or noncompaction)) | 0 |
| S40 | title(cardiomyopath* N/3 (non-compaction or noncompaction)) | 0 |
| S41 | title(congenital N/2 (heart? or cardia*) N/2 block*) | 0 |
| S42 | MAINSUBJECT.EXACT("Nursing Education") | 6,036 |
| S43 | MAINSUBJECT.EXACT("Continuing Education") | 5,475 |
| S44 | title((((nurs* or clinical) N/4 (educat* or train* or learn* or knowledg*)) or professional development or continuing educat* or ((learning or education* or knowledge) N/4 (need* or gap* or requirement* or deficien* or improv*)) or competenc* or (skill* N/3 train*) or (understanding N/3 improv*))) OR abstract((((nurs* or clinical) N/4 (educat* or train* or learn* or knowledg*)) or professional development or continuing educat* or ((learning or education* or knowledge) N/4 (need* or gap* or requirement* or deficien* or improv*)) or competenc* or (skill* N/3 train*) or (understanding N/3 improv*))) | 255,348 |
| S45 | [S1] OR [S2] OR [S3] OR [S4] OR [S5] OR [S6] OR [S7] OR [S8] OR [S9] OR [S10] OR [S11] OR [S12] OR [S13] OR [S14] OR [S15] OR [S16] OR [S17] OR [S18] OR [S19] OR [S20] OR [S21] OR [S22] OR [S23] OR [S24] OR [S25] OR [S26] OR [S27] OR [S28] OR [S29] OR [S30] OR [S31] OR [S32] OR [S33] OR [S34] OR [S35] OR [S36] OR [S37] OR [S38] OR [S39] OR [S40] | 32 |
| S46 | [S42] OR [S43] OR [S44] | 259,733 |
| S47 | [S45] AND [S46] | 5 |

**Scopus Search Strategy**

| **#** | **Query** | **Results from 15 Apr 2026** |
| --- | --- | --- |
| 1 | (((congenital or neonat* or pediatric or structural or cyanotic or acyanotic) W/3 (heart or cardiac or coronary or septal* or aortopulmonary or aorticopulmonary or atrial or ventricular or intraventricular) W/3 (defect* or disease* or abnormal* or anomal* or malform* or condition* or care)) or (single ventricle defect* or biventricular defect*)) | 154,207 |
| 2 | ((atrial or ventricular or atrioventricular) W/3 septal W/3 defect) | 44,895 |
| 3 | (pulmonary W/2 (atresia or regurgitation or stenosis or prolapse)) | 29,388 |
| 4 | (tricuspid W/2 (atresia or regurgitation or stenosis or prolapse)) | 36,042 |
| 5 | Eisenmenger syndrome | 1,946 |
| 6 | (neonatal W/3 (cardiopath* or cardiomyopath*)) | 233 |
| 7 | (aort* W/2 (coarctation* or narrow* or stenosis)) | 83,635 |
| 8 | (bicuspid W/3 aort* W/3 valve*) | 9,920 |
| 9 | (persistent W/3 truncus W/3 arteriosus) | 1,245 |
| 10 | (truncus W/3 arteriosus W/3 communi*) | 225 |
| 11 | (endocardi* W/3 cushion* W/3 defect*) | 1,804 |
| 12 | (atrioventricular W/3 (canal* or cushion*)) | 2,820 |
| 13 | (aortopulmonary W/2 window*) | 915 |
| 14 | (common W/3 arter* W/3 trunk*) | 1,301 |
| 15 | (persistent W/3 ostium W/3 primum*) | 19 |
| 16 | (patent W/3 oval? W/3 foramen*) | 13,024 |
| 17 | (lutembacher* W/2 syndrome*) | 230 |
| 18 | (double W/3 outlet* W/3 right W/3 ventricle*) | 4,986 |
| 19 | (taussig W/3 bing W/3 anomal*) | 284 |
| 20 | (hypoplastic W/3 left W/3 heart* W/3 syndrome*) | 8,415 |
| 21 | (fallot* W/2 (tetralog* or tetrad* or syndrome*)) | 22,327 |
| 22 | (transposition W/3 great W/3 (arter* or vessel*)) | 14,993 |
| 23 | (levotransposition* W/3 great W/3 (arter* or vessel*)) | 10 |
| 24 | (levo W/2 tga*) | 12 |
| 25 | L-TGA | 85 |
| 26 | (dextrotransposition* W/3 great W/3 (arter* or vessel*)) | 70 |
| 27 | (dextro W/2 tga*) | 23 |
| 28 | D-TGA | 560 |
| 29 | (congenital* W/3 corrected W/3 (transposition* or tga*)) | 1700 |
| 30 | CC-TGA | 70 |
| 31 | (left W/3 right W/3 shunt*) | 12,957 |
| 32 | (paten* W/3 atrioventricular W/3 canal*) | 29 |
| 33 | (cyanotic W/3 (cardia* or heart?) W/3 (disease* or defect*)) | 137 |
| 34 | (myocardial W/2 bridg*) | 2346 |
| 35 | (single W/2 heart? W/2 ventricle?) | 4 |
| 36 | ((monoventricular or univentricular) W/2 heart?) | 470 |
| 37 | (cor W/2 monoventricolare) | 0 |
| 38 | (cor W/2 triloculare W/4 (biatrium or biabriatum or biatriorum)) | 5 |
| 39 | (ventricular W/2 (non-compaction or noncompaction)) | 3907 |
| 40 | (cardiomyopath* W/3 (non-compaction or noncompaction)) | 1304 |
| 41 | (congenital W/2 (heart? or cardia*) W/2 block*) | 25 |
| 42 | TITLE-ABS-KEY ( ( ( ( nurs* OR clinical ) W/4 ( educat* OR train* OR learn* OR knowledg* ) ) OR professional development OR continuing educat* OR ( ( learning OR education* OR knowledge ) W/4 ( need* OR gap* OR requirement* OR deficien* OR improv* ) ) OR competenc* OR ( skill* W/3 train* ) OR ( understanding W/3 improv* ) ) ) | 252,840 |
| 43 | 1 OR 2 OR 3 OR 4 OR 5 OR 6 OR 7 OR 8 OR 9 OR 10 OR 11 OR 12 OR 13 OR 14 OR 15 OR 16 OR 17 OR 18 OR 19 OR 20 OR 21 OR 22 OR 23 OR 24 OR 25 OR 26 OR 27 OR 28 OR 29 OR 30 OR 31 OR 32 OR 33 OR 34 OR 35 OR 36 OR 37 OR 38 OR 39 OR 40 or 41 | 324,091 |
| 44 | 42 AND 43 | 330 |
