# Supplementary figures and images for: Identifying the educational needs of nurses caring for adults with congenital heart disease: A scoping review protocol
Source: PLoS One. 2026 Jul 10;21(7):e0343891. doi: 10.1371/journal.pone.0343891 (PMC13353937; doi:10.1371/journal.pone.0343891)










Supplement: S3 File — (DOCX) [file pone.0343891.s004.docx]
